# Investigation of the role of sulfide oxidation in the gill-associated microbiota of freshwater mussel *Limnoperna fortunei*

**DOI:** 10.3389/fmicb.2025.1671425

**Published:** 2025-10-13

**Authors:** Yu Peng, Duanyi Huang, Juechun Li, Xiaoxu Sun, Qifan Zhang, Ruijian Zhang, Rui Yang, Baoqin Li, Tianle Kong, Zhiming Xiong, Ying Huang, Zhibing Chang, Yuming Su, Yuming Shang, Muhammad Usman Ghani, Yingcai Wang, Weimin Sun

**Affiliations:** ^1^Changjiang Basin Ecology and Environment Monitoring and Scientific Research Center, Changjiang Basin Ecology and Environment Administration, Ministry of Ecology and Environment, Wuhan, China; ^2^Hubei Provincial Key Laboratory for Basin Ecology Intelligent Monitoring-Prediction and Protection, Wuhan, China; ^3^National-Regional Joint Engineering Research Center for Soil Pollution Control and Remediation in South China, Guangdong Key Laboratory of Integrated Agro-environmental Pollution Control and Management, Institute of Eco-environmental and Soil Sciences, Guangdong Academy of Sciences, Guangzhou, China; ^4^College of Environmental Science and Engineering, Hunan University, Changsha, China; ^5^China South-to-North Water Diversion Middle Route Corporation Limited, Beijing, China

**Keywords:** *Limnoperna fortunei*, gill-associated bacteria, microbial diversity, sulfide oxidation, water diversion projects

## Abstract

**Introduction:**

*Limnoperna fortunei* is a notable invasive freshwater species, altering structure and function of natural and engineered aquatic ecosystems. The host-associated microbiomes play a critical role in the survival and thriving of *L. fortunei*, with the gill-associated microbiomes being particularly significant due to their involvement in filter feeding, nutrient metabolism, and symbiosis. However, research on microbiomes associated with *L. fortunei* remains limited, and studies specifically focusing on gill-associated microbiota are scarce, leaving a significant gap in our understanding of their ecological roles.

**Methods:**

In this study, gill-associated bacterial communities of the *L. fortunei* were compared with their surrounding water microbial populations in the largest water diversion projects (the Middle Route of the South-to-North Water Diversion Project) to elucidate their environmental adaptations and potential contribution to their hosts. Analyses included assessing bacterial diversity and composition, conducting Neutral Community Model (NCM) analysis to explore community assembly processes, constructing an environmental-microbial co-occurrence network to identify key environmental factors, and performing metagenomic analysis of gill samples to investigate functional genes.

**Results:**

Significant variations were observed in bacterial diversity and composition between gills and surrounding water. Sulfur oxidizing bacteria *Pirellula*, *SM1A02*, and *Roseomonas* were significantly enriched in gill-associated microbiota. Neutral community model (NCM) analysis unveiled that the assembly of gill microbial communities was primarily governed by stochastic processes, constrained by determined processes. Moreover, environmental-microbial co-occurrence network identified reduced sulfur as the key factor shaping the composition of bacterial communities. Metagenomic binning of gill samples further revealed that metagenome assembled genomes associated with *Pirellula* within the phylum Planctomycetota contained functional genes related to sulfide oxidation and resistant to oxidative stress.

**Discussion:**

This study provides systematic insights into the microbial community diversity, assembly patterns, and functional characteristics of *L. fortunei* gill-asscociated microbiota, contributing to a mechanistic understanding of their ecological roles.

## 1 Introduction

Biological invasions pose one of the most significant human-induced threats to freshwater ecosystems by disrupting ecological balance, reducing biodiversity, and impairing ecosystem functions (Banha et al., 2022; Reid et al., 2019). The freshwater bivalve *Limnoperna fortunei* (the golden mussel) has emerged as a notable invasive species across various global freshwater ecosystems (Zhang J. et al., 2022). Its extensive distribution and formidable invasive capabilities have led to a series of negative impacts on local ecological environments and economic development (Boltovskoy et al., 2009). Several biological characteristics are responsible for its high invasiveness, including rapid growth, short sexual maturity maturation, and high reproduction intensity (Liu et al., 2024). In addition, it could survive under a wide range of environmental stresses, such as high contamination level, low pH, high calcium concentration, low dissolved oxygen content, and strong current (Boltovskoy et al., 2006).

Beyond these intrinsic traits, human activities also played a pivotal role in the global spread of the *L. fortunei*. The species has been found attached to numerous water conservancy and hydropower projects, including inter-basin water diversion projects (Boltovskoy et al., 2015). The concrete-lined surface offer an ideal habitat and therefore vulnerable for the attachment and aggregation of the *L. fortunei* (Xu et al., 2015). Once these mussels attach to the solid surface using their byssal threads, they can aggregate to form high population densities, leading to biofouling problems (Bonel et al., 2013). Such biofouling not only causes damage to the structural integrity, but also results in water quality degradation (Yao et al., 2017; Zhang R. et al., 2022). As the largest water transfer project in the world, the Middle Route of the South-to-North Water Diversion Project (MRSNWDP), inevitably has suffered a certain degree of *L. fortunei* attachment (Sun et al., 2023). To strengthen the control of the *L. fortunei*, it is critical to elucidate the behavior and survival strategies of the *L. fortunei*.

Previous research on the *L. fortunei* has primarily focused on the impact of environmental factors on its growth and reproduction (Lazado and Voldvik, 2020; Oliveira et al., 2011). However, the role of its associated microbiome, an non-separatable factor that contributes to its growth, has often been overlooked, despite its potential integral contribution to host physiology and environmental adaptation. As a filter-feeding bivalve, *L. fortunei* processes large volumes of water, inevitably capturing and ingesting diverse environmental microorganisms (Zhang J. et al., 2022). These microbiomes colonize multiple host tissues including the gut, mantle, and gills, where they facilitate critical functions such as nutrient acquisition, toxin detoxification, and stress resistance (Antunes et al., 2010; Lawson et al., 2022). Existing studies have shown that symbiotic microorganisms may play a key role in the ecological adaptability and survival success of mussels (Duperron et al., 2006; Ponnudurai et al., 2020). Although a significant number of genes involved in nutrient transport, stress resistance, and immune recognition were identified from symbiotic microbiota, most of these studies have focused on marine systems (Distel et al., 1995; Petersen et al., 2011). For instance, the gills of the deep-sea hydrothermal vent mussel (*Bathymodiolus thermophilus*) are inhabited by microbial symbionts that oxidize reduced sulfur and fix carbon, thereby providing energy support for the mussel’s growth (Patra et al., 2022). In contrast, research on *L. fortunei* associated microbiome remains limited, with even less attention paid to gill-associated microbiota specifically.

To clearly elucidate the ecological roles of gill-associated bacteria, it is essential to investigate the interactions metabolisms under the biogeochemical context in which these interactions occur. The MRSNWDP transfers water while simultaneously conveying nutrients, including sulfur (Li et al., 2017; Tong et al., 2022). Due to the sustained sulfur influx, sulfur cycling is an important biogeochemical process in MRSNWDP. Recent studies highlight the prevalence of a “cryptic sulfur cycle” in freshwater systems (Reese et al., 2014; Zhou et al., 2025), wherein microbial-mediated sulfate reduction and subsequent sulfide oxidation occur in tight spatial or temporal coupling, maintaining low ambient sulfide concentrations. Notably, the growth of *L. fortunei*, especially in the adult stage, consumes a certain amount of dissolved oxygen in the water (Zhang R. et al., 2022). Their high-density aggregation may affect local oxygen and potentially trigger sulfate reduction processes. While sulfide toxicity could limit mussel survival, *L. fortunei*’s remarkable adaptability suggests they have developed specific survival strategy under such circumstances. Whether the gill-associated bacteria of *L. fortunei* are involved in sulfur oxidation and affect hosts development is a question worthy of in-depth exploration.

Collectively, although existing research has made certain progress in understanding the invasion mechanisms and related ecological impacts of the *L. fortunei*, there are still many unknowns regarding the interactions between the *L. fortunei* and its host-associated bacteria in freshwater environments. In this study, tissues of gills of mussels were targeted due to their important functions in filter feeding, nutrient metabolism. The gill-associated microbiomes and surrounding water column microorganisms in MRSNWDP were subjected to 16S rRNA amplicon sequencing and metagenomic sequencing. The aims of this study were to (i) demonstrate the structure and diversity of gill-associated microbiota in *L. fortunei*; and (ii) investigate the metabolic potentials of these gill-associated bacteria, particularly regarding sulfur cycling. The findings will enhance our understanding of *L. fortunei* associated bacteria, while providing new theoretical foundations for developing effective *L. fortunei* control strategies in invaded freshwater systems.

## 2 Materials and methods

### 2.1 Sample collection

The Henan section of the MRSNWDP plays a crucial role within the overall project, serving as both the water source area and the largest recipient of water (Chen et al., 2021). In this study, adult mussels (*L. fortunei*) and surrounding water samples were collected from four representative sites in Henan section of the MRSNWDP, including YH (34.1799986° N, 113.441640° E), XZH (34.257189° N, 113.621755° E), CHQ (34.871345° N, 113.243197° E), SBL (34.660500° N, 113.686908° E). At each site, at least five *L. fortunei* individuals and surrounding water were collected using sterile containers. All samples were immediately stored on ice and subsequently returned to the laboratory.

The mussels were temporarily maintained in refrigerator at 4.0 °C until dissection. Each mussel was aseptically opened with a sterilized scalpel. The mussels were gently washed and the gill tissues were carefully excised. The gill samples were then individually placed in RNase- and DNase-free microtubes and stored at −80 °C for further molecular analysis. For water samples, a portion was filtered through sterile membrane filters (0.22 μm pore size). The filters were stored at −80 °C for DNA extraction. Another aliquot of water was stored at −20 °C for subsequent geochemical analysis.

### 2.2 Geochemical analysis

Water samples were filtered through a 0.22 μm filter membrane, and water-soluble NO_3_^−^, SO_4_^2−^ (WSS), and reduced sulfur (WSRS) were determined using an ion chromatograph (IC, Thermo Scientific, United States). Water-soluble N_NH_4_^+^ was measured using a full-wavelength microplate reader (Multiskan Sky, Thermo Fisher Scientific, United States) (Yang et al., 2022). Total nitrogen (TN) and total sulfur (TS) were estimated from unfiltered samples yby ion chromatography (ICS-600, Thermo Fisher, United States) after digestion (Colina and Gardiner, 1999).

### 2.3 DNA sequencing and analyses

DNA of water samples were extracted using a DNeasy PowerSoil kit (Qiagen, Germany), following the manufacturer’s protocol. In addition, the gills of *L. fortunei* were separated using a sterile scalpel. Subsequently, the gills were frozen in liquid nitrogen and ground using a pestle. DNA of gill-associated microorganisms were extracted from pulverized gills using the DNeasy PowerSoil kit (Qiagen, Germany) following the manufacturer’s instructions. To analyze the microbial communities in each compartment, the 515F/806R primer set (515F: 5’-GTGCCAGCMGCCGCGGTAA-3′, and 806R: 5’-GGACTACVSGGGTATCTAAT-3′) was used to target the V4 hypervariable region of the 16S rRNA gene (Sun et al., 2024). The resulting amplicons were barcoded, pooled, and sequenced using the Illumina MiSeq System (Personal Biotechnology Company, Shanghai, China).

The sequencing libraries were processed in QIIME2 (v. 2024.2) (Bolyen et al., 2019). Briefly, the primers were trimmed, the sequences (trimmed) were filtered to obtain low-quality reads, and chimeras were removed. The high-quality sequences were grouped into amplicon sequence variants (ASVs) using DADA2 (Callahan et al., 2016; Kong et al., 2024) and then searched against the SILVA (132) database for taxonomic assignment (Quast et al., 2013). The ASVs were imported into R package “phyloseq” (v1.26.1) for downstream analysis (Mcmurdie et al., 2013). The alpha and beta diversities were calculated using rarified libraries in R package “phyloseq” (v1.26.1). PERMANOVA analysis to compare statistical significance between groups was performed using the R package “vegan” (v2.6–4) (Oksanen et al., 2010). The R package “ggplot2” (v3.4.2) was used to visualize the results (Villanueva and Chen, 2019). The co-occurrence networks for biotic and env-bio interactions were computed using the R package “ggClusterNet” (v0.1.0) with parameters “N = 0, r = 0.6, *p* = 0.05, method = pearson” (Wen et al., 2022) and visualized in Gephi (v0.10.1) (Newman, 2006). The neutral community model (NCM) was calculated using R package “vegan” and visualized in “ggplot2.”

### 2.4 Metagenomic sequencing and analysis

To identify and characterize the metabolic potential of keystone taxa, one water and one gill samples were sequenced for metagenome analysis using the Illumina MiSeq System at the Personal Biotechnology Company (Shanghai, China). Trimmomatic (v0.36) was used to trim and quality control the raw reads (Bolger et al., 2014). To eliminate host interference, the reference genome sequence of the host *L. fortunei* was retrieved from the National Center for Biotechnology Information (NCBI) database. Subsequently, the retrieved *L. fortunei* genome was indexed using Bowtie2 (v2.3.5.1) with default parameters optimized for short-read alignment. The quality-controlled clean reads were then aligned to the pre-indexed *L. fortunei* genome using Bowtie2 (v2.3.5.1) with the parameter set ‘--end-to-end --very-sensitive’. The remaining reads were retained for subsequent metagenomic analyses, as they were verified to be predominantly of microbial origin.

After removing host contamination, open reading frames (ORFs) were predicted on assembled contigs using Prodigal (version 2.6.3). The predicted ORFs were processed with CD-HIT (version 4.8.1) to create a non-redundant gene set. We then quantified the relative abundances of genes associated with sulfur oxidation and sulfate reduction using Salmon (version 1.10.3). The abundance of each gene was normalized to Reads Per Million (RPM) to account for differences in sequencing depth across samples.

Metagenome binning was performed to recover microbial genomes from metagenomic data according to the protocol described (Chen et al., 2024). The filtered reads were assembled using Megahit (v1.2.9) (k = 21–121, step = 10) (Li et al., 2015). The contigs assigned to eukaryotes were removed using Blobtools2 (v1.1.1) (Laetsch and Blaxter, 2017). The filtered libraries were individually binned into metagenome assembled genomes (MAGs) using MetaWRAP (v1.3) (Uritskiy et al., 2018). The quality of MAGs was estimated using CheckM (v1.1.2), and only MAGs with completeness > 50% and contamination < 10% were retained for downstream analysis (Parks et al., 2015). The Genome Taxonomy Database (GTDB) was used to assign the taxonomic affiliations of the retrieved MAGs (Parks et al., 2018). The functional genes of the retrieved MAGs were annotated using KofamKOALA against the KEGG database (Aramaki et al., 2019). This study’s 16S rRNA and metagenomic sequencing data were submitted to GenBank with the accession number PRJNA1226116.

## 3 Results

### 3.1 Geochemical analysis

We sampled the *L. fortunei* samples from 4 sites within the MRSNWDP and characterized geochemical conditions of corresponding surrounding water samples, including the concentration of the total nitrogen (TN), total sulfur (TS), dissolved nitrate (NO_3_^−^), ammonium (NH_4_^+^), water-soluble SO_4_^2−^ (WSS), and water-soluble reduced sulfur (WSRS) (Supplementary Figure S1). The amount of total sulfur in the MRSNWDP fluctuated in the range of 21.59**–**31.23 mg/L. Notably, in four sampling sites, the proportion of WSRS in the TS was substantial, as high as 23.10–71.18%. The average value of TN and NH_4_^+^ in river water was 1.030 and 0.039 mg/L, respectively.

### 3.2 Structure and diversity of the gill-associated microbial communities

**Figure 1:**
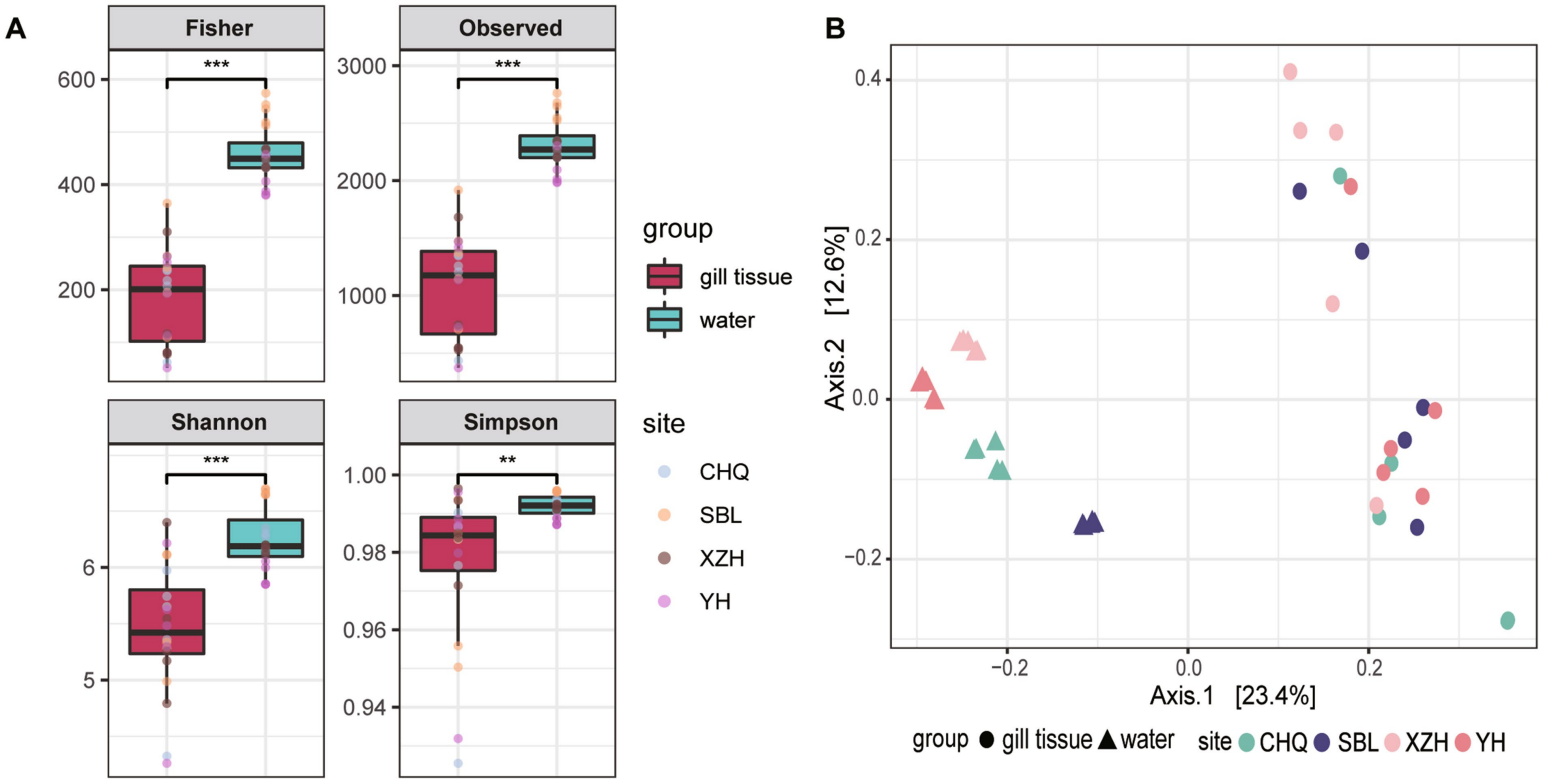
Alpha diversity indexes including Observed species, Shannon, Simpson, and Fisher of bacterial communities with water column and gill tissue **(A)**. The PCOA plot of beta diversity measured as Bray-Curtis distances for microbial communities from different habitats and sampling sites **(B)**.

16S rRNA sequencing was conducted to determine the bacterial structure and diversity of microbial community in *L. fortunei* gills and surrounding river water. The alpha diversity indices, including Observed species, Shannon, Simpson, and Fisher, were calculated (Figure 1A). Significantly elevated alpha diversities were observed in the river water comparing to the microbial community inhabiting mussel’s gill. For example, the Observed species index of the water microbiota was 2,308, on average, while 1,069 of gills microbiota. Similarly, the Shannon index was significantly higher in the river water microbial community compared with that of the gill-associated bacterial community (Shannon index: 6.25 ± 0.27 vs. 5.44 ± 0.56, *p* < 0.001).

Principal Coordinates Analysis (PCoA) based on Bray–Curtis dissimilarity was performed to visualize the beta diversity differences between the gill-associated bacteria and river water microbial communities (Figure 1B). The first two principal coordinates explained 23.4 and 12.6% of the total variance, respectively. The PCoA plot clearly separated the gill samples from the river water samples, revealed significant differences in community composition between mussel gill bacterial microbiota and bacteria suspended in the water column. Notably, the water microbial community exhibited significant clustering based on site difference.

**Figure 2:**
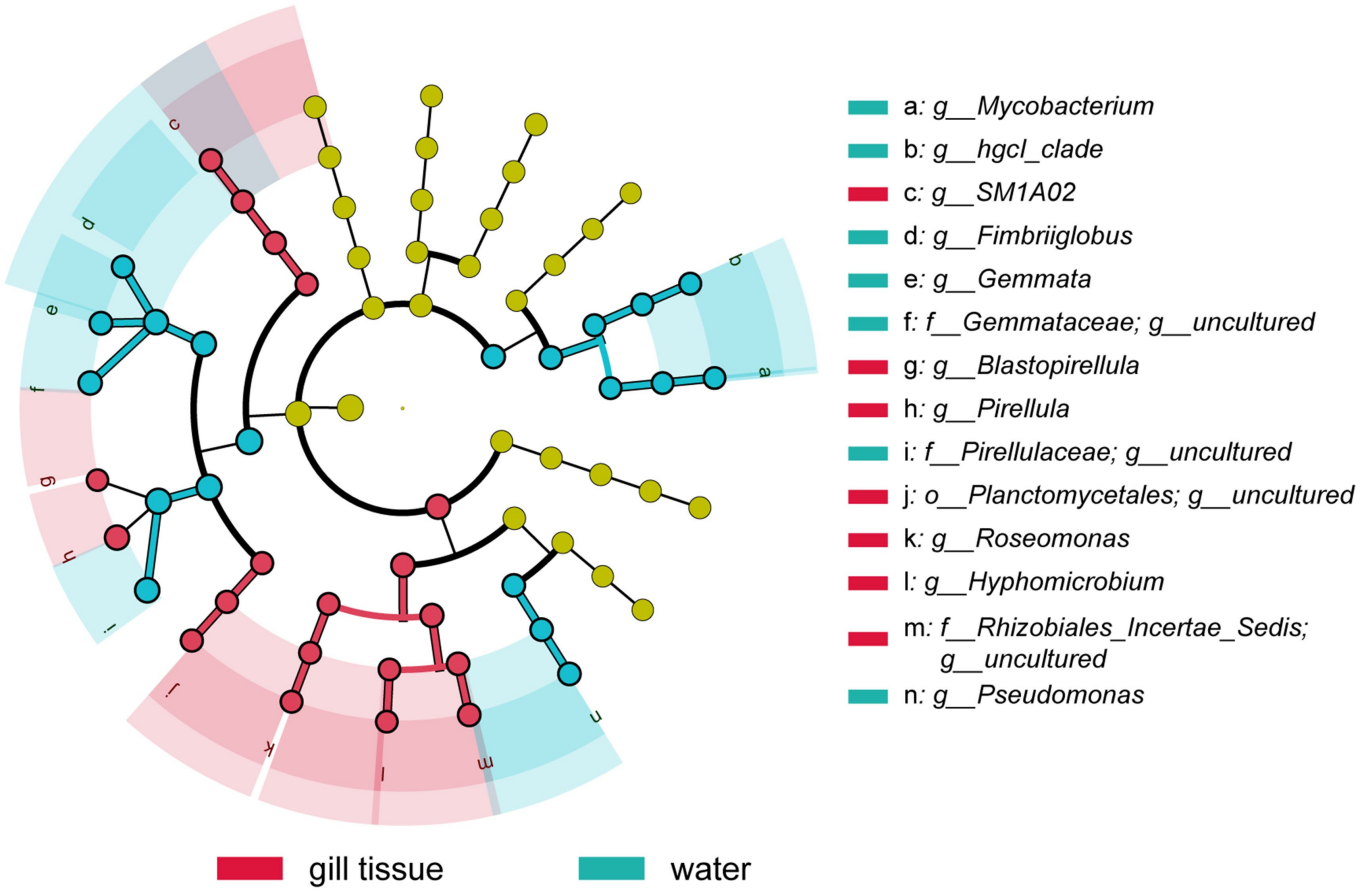
The linear discriminant analysis effect size (LEfSe) analysis at species level of bacterial communities (with LDA score >3.1 and *p* < 0.05) among water microbiota and gill-associated microbiota of golden mussel.

Planctomycetota and Proteobacteria were the dominant phyla of the gill microbial communities across all samples, which accounted for 27.44 and 32.68% of the total sequences (Supplementary Figure S2). *Pirellula* was the most abundant genus, with a relative abundance of 8.8%, followed by Cyanobium_PCC-6307 (2.2%), JG30-KF-CM45 (1.8%), SM1A02 (1.8%), *Roseomonas* (1.4%), and *Stenotrophobacter* (1.3%) in gill-associated bacterial community (Supplementary Figure S3). Among them, *Pirellula*, SM1A02, and *Roseomonas* were significantly enriched in gills microbiota, with higher relative abundance compared with surrounding river water, revealed by the LEfSe analysis (Figure 2).

### 3.3 Relative importance of neutral processes in community assembly

**Figure 3:**
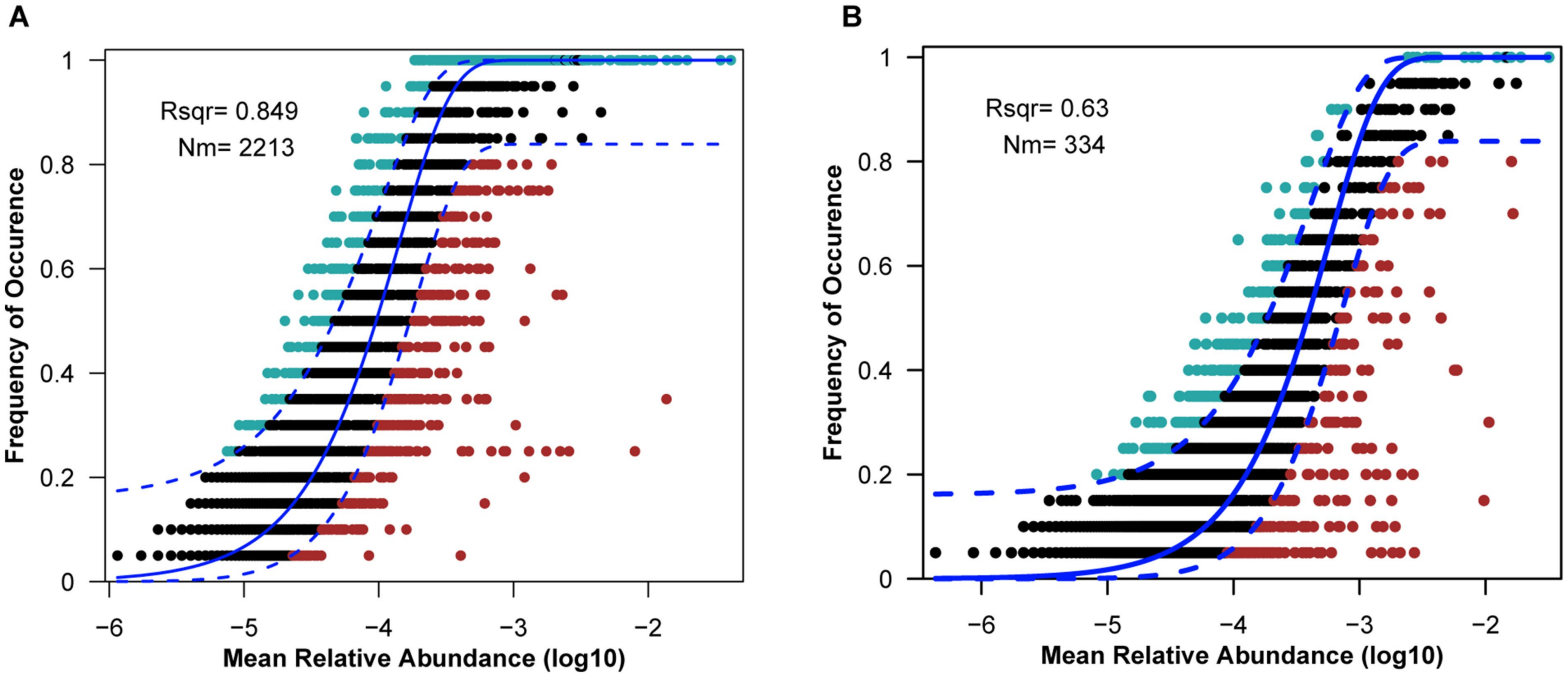
Fit of the neutral community model (NCM) of community assembly of water samples **(A)** and gill samples **(B)**. Solid blue lines represent the best fit of the neutral community model, dashed blue lines represent the 95% confidence interval around the model prediction, ASVs that occur within the predicted range are shown in black, and ASVs that occur more (blue) or less (red) than predicted by the NCM shown in different colors. Rsqr (R^2^) represents the fit degree of the model, and Nm represents the product of community size and migration times.

Neutral community model (NCM) was used to investigate the microbial community assembly process. The NCM explained 63.0 and 84.9% of the microbial communities of *L. fortunei* gills and surrounding water column, respectively (Figure 3), indicating that stochastic processes (e.g., passive dispersal, ecological drift) played a dominant but not exclusive role in community assembly. The significantly lower explanatory power (*R*^2^) and migration rate (Nm) in gill communities compared to water communities (*R*^2^ = 63.0% vs. 84.9%; Nm = 334 vs. 2,213) strongly suggests that deterministic processes exert a greater influence on structuring the gill-associated microbiota than the water column community.

### 3.4 Impact of the geochemical conditions

**Figure 4:**
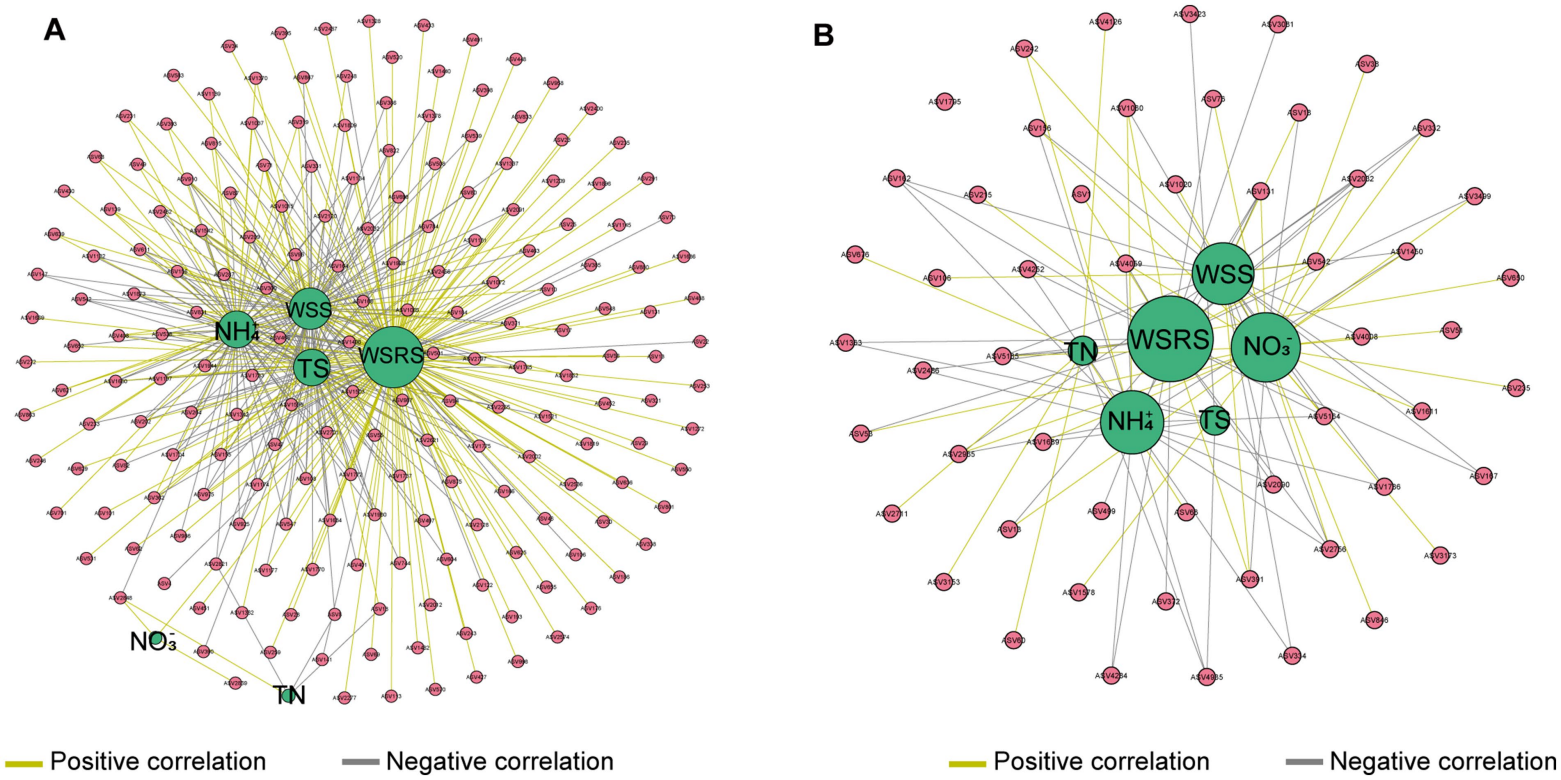
Co-occurrence network demonstrates interactions between geochemical parameters and abundant bacterial taxa within river water **(A)** and mussel’s gill **(B)**. The connection indicates a strong (|r| > 0.6) and a significant (*p* < 0.05) Pearson’s correlation. The size of each node is proportional to the number of connections (i.e., degree).

The environmental-microbial co-occurrence network was further constructed to explore the relationships between environmental factors and the microbial community structure. According tothe co-occurrence network, the geochemical parameters exerted 439 pairs of strong (Pearson correlation > 0.6) and significant (*p* < 0.05) connections on 193 microbial populations suspended in the river water (Figure 4A). In the gill-associated microbiota, 54 microbial populations were strongly impacted by the geochemical parameters and generated 164 pairs of connections (Figure 4B). WSRS was identified as a key environmental factor significantly influencing both the gill-associated bacteria and river water microbial communities.

### 3.5 Characterization of the functional potentials of gill-associated bacteria

**Figure 5:**
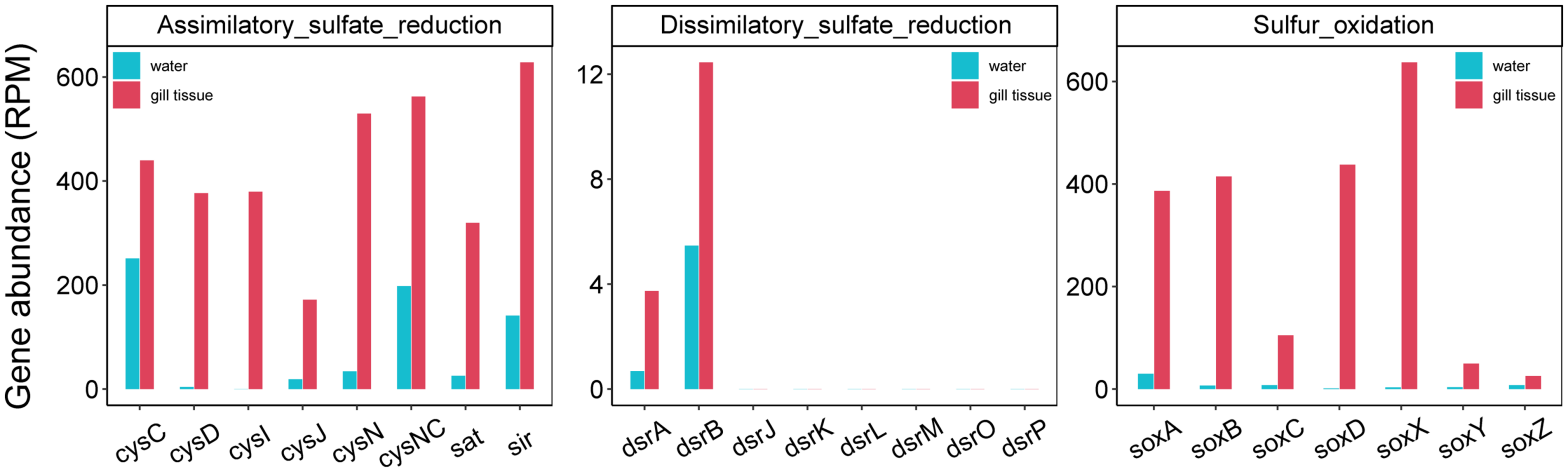
Relative abundances of genes associated with sulfur oxidation and sulfate reduction.

Given the critical role of reduced sulfur compounds in shaping gill-associated microbiota composition, we conducted a focused investigation of sulfur cycling-related genes, including SOX (sulfur oxidation) systems, dissimilatory sulfate reduction and oxidation, and assimilatory sulfate reduction. The relative abundances of the genes related to sulfur cycle were significantly more abundant in the gill-associated bacteria than those in the corresponding river water microorganisms (Figure 5). The relative abundances of sulfur cycle-related genes were significantly higher in gill-associated bacteria than in the corresponding river water microbial community (Figure 5). All gene abundances were normalized to Reads Per Million mapped reads (RPM) to account for inter-sample differences in sequencing depth. For instance, the *soxB* gene (a key marker of the SOX system) in gill-associated microbiota exhibited an RPM of 414.69, representing a 24.4-fold enrichment compared to that in water. Notably, the abundance of *dsrA* (a critical gene mediating dissimilatory sulfate reduction and oxidation) was substantially lower than that of *soxB*, with respective RPM values of 3.74 and 414.69, respectively.

**Figure 6:**
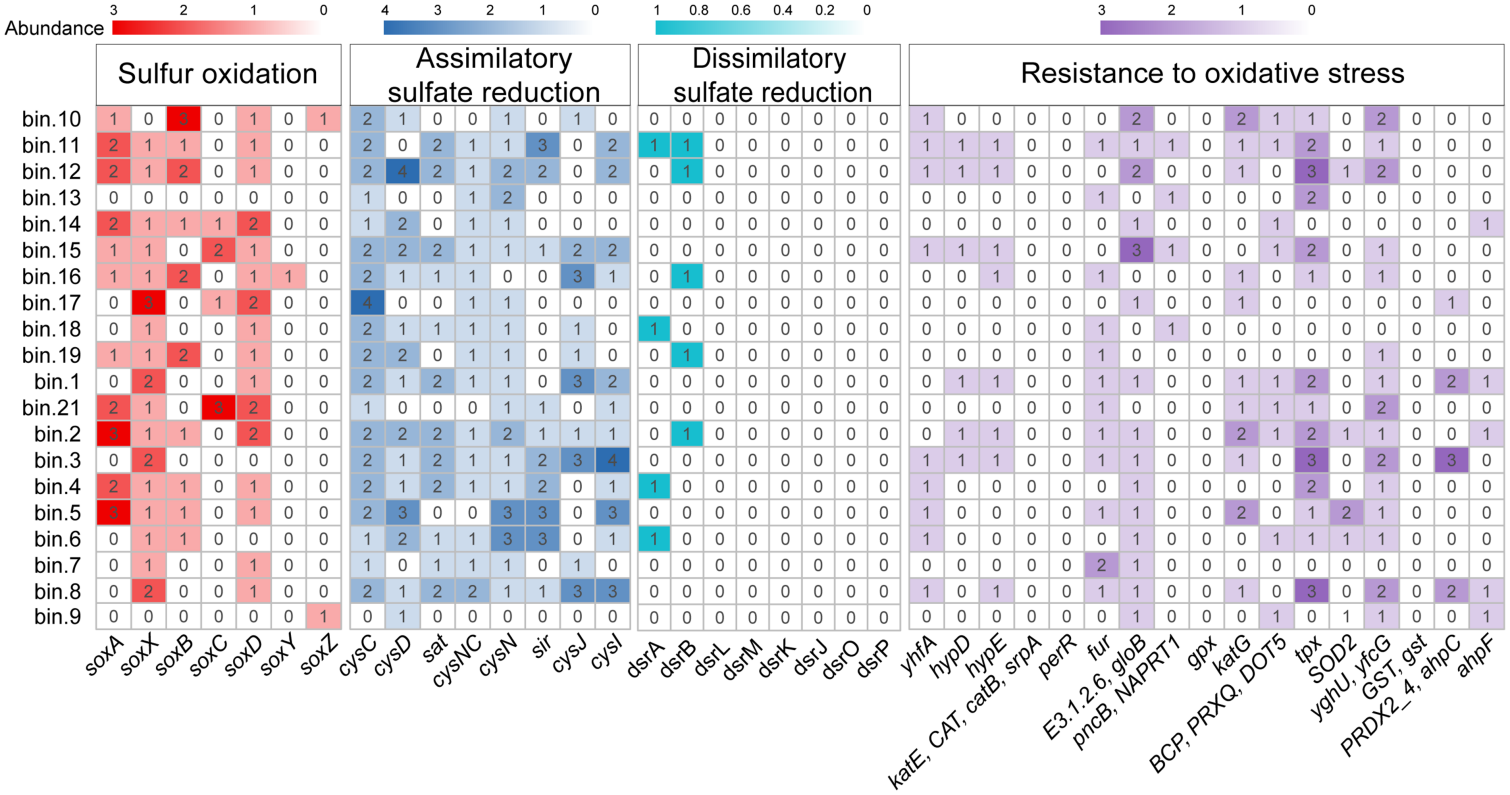
Heatmap of genes abundance encoding sulfur oxidation, sulfate reduction and resistance to oxidative stress detected in the MAGs.

Metagenomic-binning was performed to recover the MAGs of gill-associated microbiomes of the *L. fortunei* for further verification of their metabolic potentials. Metagenomic binning reconstructed 20 MAGs (completeness > 50% and contamination < 10%) that represented 7 microbial phyla, including 9 MAGs assigned to the Planctomycetota (Supplementary Figure S4). MAG.2 was classified as a member of the genus *Pirellula*, the most abundant genus in gills microbiota (Supplementary Table S1). Functional gene annotation of these MAGs revealed a diverse range of metabolic capabilities involved in sulfur oxidation, sulfate reduction and resistant to oxidative stress (Figure 6). Most of the MAGs affiliated to Planctomycetota (MAG 2, 4, 5, 6, 11, 12, 14, 16) contained the *soxB* gene associated with sulfur oxidation. Gill-associated MAGs also contained multiple genes involved in antioxidant defense, such as *yhfA*, *hydD*, *hydE*, *fur*, *gloB*, *katG*, *tpx*, *SOD2*, and *yfcG*.

## 4 Discussion

Gill-associated microbiomes of *L. fortunei* may play an important ecological role to promote the growth of the host, which may then thrive the population of *L. fortunei*. Therefore, it is urgent to investigate the microbial diversity and compositions of gill-associated microbiota. However, such information was overlooked. In this study, the microbial community and metabolic potential of gill-associated microbiota of *L. fortunei* was investigated and analyzed.

### 4.1 Community structure and assembly mechanism of gill-associated microbiota

The microbial communities associated with *L. fortunei* gills exhibited distinct patterns compared to those in the surrounding water revealed by beta diversity analysis (Figure 1B). This divergence arises from a dynamic interplay of stochastic processes and deterministic forces, with neither exclusively governing community assembly.

Neutral community modeling (NCM) reveals a substantial role for stochasticity, explaining 63.0% of the variation in gill-associated microbial composition (Figure 3B), which indicated that a large fraction of gill-associated taxa was recruited through random dispersal from the surrounding water. This pattern is linked to the filter-feeding behavior of *L. fortunei*, which processes large volumes of water and passively captures planktonic microbes (Darrigran, 2002). Colonization of the gill surface is partially driven by stochastic events, including random encounters with suitable attachment sites and ecological drift in small populations. Comparisons with the water column, where NCM explained 84.9% of variation, further highlighted the influence of unconstrained dispersal in free-living communities.

Despite the prominence of stochasticity, deterministic process (primarily host selection and environmental filtering) modulates community assembly. The lower Rsqr and Nm values in microbiota of mussel gills relative to the water column indicated host-imposed constrains on random dispersal. The gill microenvironment, characterized by physiological barriers such as epithelial mucus layers, immune recognition systems including transmembrane receptors that distinguish microbial surface molecules, and localized chemical gradients, functions as a selective filter (Lin et al., 2023; Won et al., 2003). Moreover, the alpha diversity of the gill-associated microbiota is lower than that of the water column, as only taxa with adaptations to survive in the gill microenvironment are retained, further suggesting the existence of a selective filtration process in the gills. Other freshwater mussels like *Fusconaia cerina* and *Lampsilis ornata* also actively shape their associated microbiomes through species-specific selective retention, generating communities distinct from the seston they filter (Weingarten et al., 2019). Environmental factors further refine this filtered community. Based on the geochemical condition of sampling sites within MRSNWDP, the environmental-microbial co-occurrence network analysis revealed that reduced sulfur concentration (WSRS) was the core environmental factor affecting the structure of *L. fortunei* gill-associated microbial community under local context (Figure 4B). For example, *Pirellula*, the most abundant genus in gill-associated microbiota, showed a significantly positive association with WSRS, reflecting its adaptation to sulfide-rich niches (Elshahed Mostafa et al., 2007).

In the gill-associated microbiota, Planctomycetota and Proteobacteria were the dominant phyla, which is consistent with previous studies on the microbiomes of other freshwater mussel species, such as *Fusconaia cerina* and *Amblema plicata* (Cotten, 2021; Weingarten et al., 2019). *Pirellula* along with SM1A02 and *Roseomonas* was significantly enriched in the gill-associated microbiota compared to the surrounding water (Figure 2). These microbiomes are commonly present in sulfidic environments, such as phycosphere of the algal bloom, thermal spring and mine tailing. *Pirellula* has a close connection with diatom bloom, and has been reported to obtains energy from cleavage of sulfated polymers produced by algae (Morris et al., 2006; Schlesner et al., 2004). Similarly, bacteria associated with *SM1A02* have been detected in high abundance within the phycosphere of the bloom-forming cyanobacterium *Raphidiopsisraciborskii* (Vico et al., 2021). Moreover, *Roseomonas* was identified as abundant genus and may play a key role in phototrophic nitrogen fixation in mine tailing (Li et al., 2024a, 2024b).

In summary, the assembly of *L. fortunei*’s gill-associated microbiota follows a model of stochasticity constrained by determinism. Stochastic dispersal from the water column provides the initial species pool. Host selection and environmental filtering then act as directional forces to shape the final community. This balance explains why gill-associated communities differ from the water column while retaining a strong stochastic signature and underscores the complexity of host-microbe-environment interactions in shaping symbiotic communities of invasive freshwater bivalves.

### 4.2 S-oxidizing potential of gill-associated bacteria

The functional metagenomic analyses revealed a pronounced enrichment of genes associated with sulfur oxidation, particularly the key marker *soxB*, within the gill-associated microbial community compared to the surrounding water column (Figure 5). This genetic potential was further supported by its prevalence in metagenome-assembled genomes (MAGs) affiliated with the phylum Planctomycetota, most notably in a MAG classified as *Pirellula* (Figure 6; Supplementary Table S1), which was also among the most abundant genera enriched in gill-associated microbiota (Figure 2; Supplementary Figure S3). Members of Planctomycetota are ubiquitous aquatic bacteria involved in C, N and S cycle (Bondoso et al., 2017; Ivanova et al., 2018; Schlesner, 1994). The genus *Pirellula* within Planctomycetota, frequently observed in sulfur-rich environments and isolated from sulfide-saturated sediments (Elshahed Mostafa S. et al., 2007), is consistently linked to sulfur oxidation processes.

The enrichment of sulfur oxidation (sox) genes suggested that the oxidation of reduced sulfur compounds represents a key metabolic strategy for a significant portion of the gill-associated microbiota. This functional trait is likely a critical adaptive response to the local biogeochemical conditions. While dissolved sulfide concentrations in oxic riverine waters typically remain below detection limits, localized hypoxia microenvironments may accumulate sulfide. Such conditions arise from sustained sulfur inputs in the MRSNWDP channel (Li et al., 2017) and are exacerbated by high-density *L. fortunei* aggregations. These aggregations reduce oxygen availability through respiration and the decomposition of biodeposits (Karatayev et al., 2010). Dense *L. fortunei* colonies increase surface roughness, thus reducing flow velocity and limiting oxygen replenishment (Boltovskoy et al., 2015). Such microenvironments are conducive to sulfate reduction and the accumulation of sulfide, thereby selecting for microbial taxa capable of detoxifying and utilizing these reduced sulfur compounds.

Mussel gills are the primary tissue in contact with external sulfides (Xu et al., 2024). The capacity to perform sulfide oxidation provides a competitive advantage for colonizing the gill niche, where sulfide exposure may occur episodically. This aligns with the positive association observed between *Pirellula* and water-soluble reduced sulfur (WSRS) in the co-occurrence network (Figure 4B), indicating an environmental adaptation to sulfidic conditions. Taxa within Planctomycetota, including *Pirellula*, have been reported in sulfidic freshwater habitats, where they are hypothesized to participate in sulfur cycling (Ivanova et al., 2018).

Beyond microbial niche adaptation, sulfide oxidation potential may indirectly benefit the host. Although direct evidence of metabolite exchange is not provided by the current data, the microbial-mediated oxidation of sulfide in the immediate vicinity of the gill tissue could potentially confer an indirect benefit to the mussel. Sulfide, especially lipid soluble H_2_S, can bind tightly with the Cytochrome c oxidase in the electron transport chain, thereby suppressing the function of oxidative phosphorylation in mitochondria and causing serious physiological damage to organisms (Cooper and Brown, 2008). Therefore, a microbial community actively oxidizing sulfide could help mitigate the toxicity of this compound, potentially preventing damage to the gill epithelium and supporting host fitness in environments where sulfide might transiently accumulate. Moreover, S-oxidizing bacteria may promote the bio-degradation of sulfur-containing organic pollutant. For example, 4,4′-dichlorodiphenyl sulfide, a sulfur-containing dioxin-like pollutant widely present in aquatic environment, was predominantly metabolized through S oxidation in the freshwater mussel *Anodonta woodiana* (Zhang et al., 2018).

Collectively, the capacity of gill-associated microbiota to oxidize sulfides could enhance microbial adaption and help regulate sulfur levels in the immediate environment, which in turn might influence the mussel’s ability to survive in sulfidic conditions. This interplay may be relevant to understanding *L. fortunei*’s presence in systems like the MRSNWDP, where sulfur dynamics are amplified by human activities. Further field studies and laboratory experiments are thus needed to define the ecological roles and underlying mechanisms of these gill-associated bacteria in freshwater ecosystems.

### 4.3 Antioxidant defense potential of gill-associated bacteria

In addition to sulfur oxidation capability, the antioxidant defense potential of gill-associated bacteria is critical for the colonization and environmental adaptation of the bacterial community. Prooxidant activities are exerted by the host to kill the invading microbe by exposure to ROS, whereas antioxidant activities are used by symbiotic microbiomes to escape the host defensive measures (Soudant et al., 2013). This antioxidant capacity also indirectly supports L. fortunei’s growth by protecting *L. fortunei*’s growth-related processes and maintaining maintains redox homeostasis for bacterial metabolism, including sulfur oxidation (Valdés et al., 2008). The gills are constantly exposed to oxidative stressors, both from internal metabolic processes and external environmental factors. Reactive oxygen species (ROS) generated under these conditions can damage cellular components such as DNA, proteins, and lipids (Lazado and Voldvik, 2020; Lushchak, 2011). If left unmitigated, this damage can disrupt essential cellular functions, including those related to growth, such as cell division and protein synthesis.

In the current study, MAGs of gill-associated bacteria also contained various genes associated with resistance to oxidative stress. For example, MAG2 contained superoxide dismutase encoding gene (*SOD2*), catalase-peroxidase genes (*katG*) and thioredoxin-dependent peroxiredoxin encoding gene (*tpx*), indicated its ability involved in antioxidant defense. Superoxide dismutase (SOD) and catalase (CAT) are the primary antioxidant enzymes responsible for eliminating excess ROS (Sharma et al., 2012). During this process, ROS is transformed to H_2_O_2_ by SOD, and H_2_O_2_ is converted to O_2_ and H_2_O by CAT (Ren et al., 2013). Gill associated bacteria might protect cell metabolism by acting as antioxidants to scavenge free radicals and ROS generated under stress, and stabilize the structure of biological macromolecules (Fernández et al., 2012).

Overall, functional gill-associated bacteria of *L. fortunei*, such as *Pirellula*, may enhance host colonization and growth through sulfur oxidation and antioxidant capabilities. These processes enable detoxification, provide additional energy, and mitigate oxidative stress, thus supporting mussel proliferation. In MRSNWDP, *L. fortunei* poses significant biofouling risks. Disrupting the symbiotic relationship between mussels and their gill-associated bacteria may offer a novel control strategy. Targeted enzymatic inhibitors or competitive microbes could be introduced to interfere with sulfur oxidation and antioxidant functions, inhibiting mussel proliferation. However, further investigation is required to assess the practical potential of such approaches.

## 5 Conclusion

In conclusion, our study provides a comprehensive analysis of the microbial communities associated *L. fortunei* gills within the MRSNWDP. We demonstrated that the gill-associated microbiota is shaped by a complex interplay of deterministic and stochastic processes, with significant contributions from host selection and environmental filtering. The prevalence of sulfur-oxidizing bacteria among gill-associated microbiota may play a crucial role in the detoxification of harmful reduced sulfur compounds and may provide additional energy for the host growth. Antioxidant capabilities of gill-associated bacteria also improve host fitness. Our findings underscore the potential impact of considering microbial symbionts in the host’s growth. Further research is needed to explore the mechanisms underlying these interactions and to develop effective strategies for managing the spread of invasive species like *L. fortunei*.

## Data Availability

The datasets presented in this study can be found in online repositories. The names of the repository/repositories and accession number(s) can be found at: https://www.ncbi.nlm.nih.gov/, PRJNA1226116.
