# Supplementary material for: Investigation of the role of sulfide oxidation in the gill-associated microbiota of freshwater mussel Limnoperna fortunei
Source: Front Microbiol. 2025 Oct 13;16:1671425. doi: 10.3389/fmicb.2025.1671425 (PMC12557512; doi:10.3389/fmicb.2025.1671425)
Supplement: Supplementary file 1 [file Supplementary_file_1.docx]

*Supplementary Information (SI) for*

**Investigation of the role of sulfide oxidation in the gill-associated microbiota of freshwater mussel *Limnoperna fortunei***

Yu Peng ^a†^, Duanyi Huang ^b,d†^, Juechun Li ^c^, Xiaoxu Sun ^b^, Qifan Zhang ^c^, Ruijian Zhang ^b^, Rui Yang ^a^, Baoqin Li ^b^, Tianle Kong ^b^, Zhiming Xiong ^a^, Ying Huang ^b^, Zhibing Chang ^c^, Yuming Su ^a^, Yuming Shang ^c^, Muhammad Usman Ghani ^b^, Yingcai Wang ^a*^, Weimin Sun ^b*^

^a^ Changjiang Basin Ecology and Environment Monitoring and Scientific Research Center, Changjiang Basin Ecology and Environment Administration, Ministry of Ecology and Environment, Wuhan, 430010, China; Hubei Provincial Key Laboratory for Basin Ecology Intelligent Monitoring-Prediction and Protection, Wuhan, 430010, China

^b^ National-Regional Joint Engineering Research Center for Soil Pollution Control and Remediation in South China, Guangdong Key Laboratory of Integrated Agro-environmental Pollution Control and Management, Institute of Eco-environmental and Soil Sciences, Guangdong Academy of Sciences, Guangzhou 510650, China

^c^ China South-to-North Water Diversion Middle Route Corporation Limited, Beijing 100038, China; Hubei Provincial Key Laboratory for Basin Ecology Intelligent Monitoring-Prediction and Protection, Wuhan, 430010, China

^d^ College of Environmental Science and Engineering, Hunan University, Changsha 410082, China

^†^These authors contributed equally to this work.

*Correspondence to:

Dr. Yingcai Wang

13 Yongqing Road, Hubei, Wuhan, China

Phone: 86- 027-82901053

Fax: 86- 027-82861430

Dr. Weimin Sun

808 Tianyuan Road, Guangzhou, Guangdong, China

Phone: 86-020-87024633

Fax: 86-020-87024123

**The Supporting Information contains 9 pages with 4 figures and 1 table.**

Supplementary Figures

Fig. S1. Geochemical parameters of water samples…………………………...…………………S4

Fig. S2. Dominant phylum in microbial community……………………………...…...…………S5

Fig. S3. The top 20 abundant genus in microbial community……………………….……………S6

Fig. S4. The phylogenetic reconstruction of MAGs……………….….…….……………………S7

Supplementary Table

Table S1. Taxonomic annotation of MAGs.…….….….……….…….……………………S8

**Fig. S1.** Geochemical parameters of water samples collected from four sampling sites within the MRSNWDP in Henan Province.

**Fig. S2.** Relative abundance of dominant phylum in microbial community within gill tissue of *L. fortunei* and surrounding river water.

**Fig. S3.** The top 20 abundant genus in microbial community within gill tissue of *L. fortunei* and surrounding river water.

**Fig. S4.** The phylogenetic reconstruction of MAGs retrieved from gill samples. The quality of the MAGs (completeness, contamination and GC content) was provided as heatmaps.

**Table S1.** Taxonomic annotation of MAGs retrieved from *L. fortunei* gills.

|  | Taxonomy |
| --- | --- |
| MAG 1 | d_Bacteria;p_Cyanobacteria;c_Cyanobacteriia;o_PCC-6307;f_Cyanobiaceae;g_CAIQIA01; |
| MAG 2 | d_Bacteria;p_Planctomycetota;c_Planctomycetia;o_Pirellulales;f_Pirellulaceae;g_Pirellula; |
| MAG 3 | d_Bacteria;p_Cyanobacteria;c_Cyanobacteriia;o_PCC-6307;f_Cyanobiaceae;g_Cyanobium; |
| MAG 4 | d_Bacteria;p_Planctomycetota;c_Planctomycetia;o_Pirellulales;f_UBA1268;g_F1-20-MAGs016;  s_F1-20-MAGs016 sp018970785 |
| MAG 5 | d_Bacteria;p_Planctomycetota;c_Planctomycetia;o_Pirellulales;f_UBA1268;g_QWOP01; |
| MAG 6 | d_Bacteria;p_Planctomycetota;c_Planctomycetia;o_Pirellulales;f_UBA1268;g_RGVT01; |
| MAG 7 | d_Bacteria;p_Actinobacteriota;c_Actinomycetia;o_Nanopelagicales;f_AcAMD-5;g_ATZT02;s_ATZT02 sp005789325 |
| MAG 8 | d_Bacteria;p_Cyanobacteria;c_Cyanobacteriia;o_PCC-6307;f_Cyanobiaceae;g_NIES-981; |
| MAG 10 | d_Bacteria;p_Bacteroidota;c_Bacteroidia;o_Chitinophagales;f_Chitinophagaceae;g_SXYR01;s_SXYR01 sp005788265 |
| MAG 11 | d_Bacteria;p_Planctomycetota;c_Planctomycetia;o_Pirellulales;f_UBA1268;g_QWPN01; |
| MAG 12 | d_Bacteria;p_Planctomycetota;c_Planctomycetia;o_Pirellulales;f_UBA1268;g_RGVT01; |
| MAG 13 | d_Bacteria;p_Actinobacteriota;c_Actinomycetia;o_Actinomycetales;f_Microbacteriaceae;g_Aquiluna; |
| MAG 14 | d_Bacteria;p_Planctomycetota;c_Planctomycetia;o_Gemmatales;f_Gemmataceae;g_REEB421; |
| MAG 15 | d_Bacteria;p_Actinobacteriota;c_Actinomycetia;o_Mycobacteriales;f_Mycobacteriaceae;g_Mycobacterium; |
| MAG 16 | d_Bacteria;p_Planctomycetota;c_Planctomycetia;o_Pirellulales;f_UBA1268;g_QWPN01; |
| MAG 17 | d_Bacteria;p_Proteobacteria;c_Alphaproteobacteria;o_Pelagibacterales;f_Pelagibacteraceae;g_Fonsibacter;  s_Fonsibacter ubiquis |
| MAG 18 | d_Bacteria;p_Chloroflexota;c_Limnocylindria;o_Limnocylindrales;f_Limnocylindraceae;g_Limnocylindrus; |
| MAG 19 | d_Bacteria;p_Actinobacteriota;c_Actinomycetia;o_Nanopelagicales;f_Nanopelagicaceae;g_MAG-120802;  s_MAG-120802 sp003569145 |
| MAG 21 | d_Bacteria;p_Verrucomicrobiota;c_Verrucomicrobiae;o_Verrucomicrobiales;f_Verrucomicrobiaceae; |
